# *Yiqi Wenyang Jiedu* prescription for preventing and treating postoperative recurrence and metastasis of gastric cancer: a randomized controlled trial protocol

**DOI:** 10.3389/fonc.2024.1326970

**Published:** 2024-07-05

**Authors:** Luchang Cao, Guanghui Zhu, Xinmiao Wang, Ziyu Kuang, Xiaotong Song, Xinyi Ma, Xiaoyu Zhu, Ruike Gao, Jie Li

**Affiliations:** ^1^ Department of Oncology, Guang’anmen Hospital, China Academy of Chinese Medical Sciences, Beijing, China; ^2^ Graduate School, Beijing University of Chinese Medicine, Beijing, China

**Keywords:** randomized controlled trial, recurrence, metastasis, traditional Chinese medicine, gastric cancer

## Abstract

**Introduction:**

Postoperative recurrence and metastasis of gastric cancer (GC) are primary factors that contribute to poor prognosis. GC recurs at a rate of approximately 70%–80% within 2 years after local treatment and approximately 90% within 5 years. “*Yang-deficient toxic node*” is the core pathogenesis of GC recurrence and metastasis. The *Yiqi Wenyang Jiedu* prescription (YWJP), a form of complementary and alternative medicine in China, is an empirical remedy to prevent postoperative recurrence and metastasis of GC. Taking the main therapeutic principles of “nourishing *Qi* and warming *Yang*, strengthening *Zhengqi*, and detoxifying” can aid in preventing the recurrence and metastasis of GC in patients during the watchful waiting period after surgery and adjuvant chemotherapy. This approach aims to enhance the quality of life of patients. However, high-quality evidence to support this hypothesis is lacking. This study will aim to investigate the efficacy and safety of YWJP to prevent and treat postoperative metastasis and GC recurrence.

**Methods:**

The study will be a multicenter, randomized, double-blind, placebo-parallel-controlled clinical trial. A total of 212 patients who completed adjuvant chemotherapy within 8 months of radical gastrectomy will be enrolled. Patients in the intervention group will receive the YWJP, whereas those in the control group will receive a placebo. The main outcome was the disease-free survival (DFS) rate 2 years after surgery. The secondary outcomes included DFS time, overall survival, annual cumulative recurrence and rate of metastasis after 1–3 years, cumulative annual survival after 1–3 years, fat distribution-related indicators, tumor markers, peripheral blood inflammatory indicators, prognostic nutritional index, symptoms and quality of life evaluation, medication compliance, and adverse reaction rate.

**Discussion:**

There is a lack of effective therapy after the completion of adjuvant therapy during the postoperative period of watchful waiting. This study will be the first randomized clinical trial to evaluate whether complementary and alternative medical interventions can effectively prevent recurrence and metastasis during the watchful waiting period after GC surgery and to provide evidence for surveillance treatment management after GC surgery.

**Clinical trial registration:**

ClinicalTrials.gov, identifier NCT05229809.

## Highlights

- Question
- Can *Yiqi Wenyang Jiedu* prescription reduce the rate of recurrence and metastasis in patients with gastric cancer during the watchful waiting period after surgery?
- Findings
- This randomized clinical trial will involve 212 patients with GC who completed adjuvant chemotherapy after surgery. The primary outcome was disease-free survival at a rate of 2 years after surgery.
- Meaning
- This ongoing clinical trial will supply the treatment management during the watchful waiting period after GC surgery and provide evidence for the effectiveness of complementary and alternative medicine in reducing recurrence and metastasis rates after GC surgery.

## 1 Introduction

Gastric cancer (GC) is a common malignant tumor of the digestive system, and according to the Global Cancer Statistics 2022, the number of new GC cases worldwide is 968,350, ranking fifth among all malignancies. The number of deaths was 659,853, ranking fifth among all cancer types (1, 2). China has the highest cancer burden worldwide, according to the National Cancer Center of China (3), and they projected that an estimated 4,824,700 new cancer cases and 2,574,200 new cancer deaths will occur in China in 2022. Among these, GC ranked fifth and third among new cases and deaths, respectively. Despite a decline in GC incidence observed in many countries, the total number of GC cases worldwide shows a slow upward trend, particularly in China (4, 5). Even more terrifying is that the global burden caused by the rising trend of GC cases is expected to increase by 62% to 1.77 million cases in 2040 (6). Studies have confirmed that various factors such as *Helicobacter pylori* infection (7), cigarette smoking (8, 9), heavy alcohol consumption (10–12), sex (13), obesity (14, 15), metabolic dysfunction (16, 17), and so on (18–26) are recognized as major risk factors for GC incidence.

Surgery is currently the preferred treatment for GC. The National Comprehensive Cancer Network guidelines (2023. V1) recommend that patients with stage pT3–4 cancer, any N, should undergo post-surgical chemoradiotherapy followed by a period of surveillance and management (27–39). Approximately 70%–80% of patients with GC relapsed within 2 years after local therapy. The median recurrence time for GC is approximately 16.8 months (40–42). Previous studies have shown that *Yang* deficiency is one of the main syndromes of GC (43), *Yang* deficiency and a low metabolic state can stimulate cancer cells to induce metabolic reprogramming and promote postoperative recurrence and metastasis of GC (44–48). Therefore, during the period between postoperative recovery and the onset of GC recurrence and metastasis, identifying effective treatment strategies is crucial and requires urgent attention.

Complementary and alternative medicine is a safe and effective potential choice that can improve the overall survival (OS) of patients with GC (49–51). Previous studies have indicated that patients with GC who experience *Qi* and *Yang* deficiencies after surgery are more susceptible to recurrence and metastasis than those with no *Qi* and *Yang* deficiencies (52, 53). The *Yiqi Wenyang Jiedu* prescription (YWJP) follows the treatment principles of nourishing *Qi* and warming *Yang*, strengthening *Zhengqi*, and detoxifying, and can improve the physical condition and alleviate clinical symptoms in patients with *Yang* deficiency (54, 55). YWJP may improve patients’ low metabolic state by regulating the tumor microenvironment and delaying or reversing recurrence and metastasis. Therefore, we plan to conduct a multicenter, randomized, double-blind, placebo-parallel-controlled clinical trial that will apply YWJP, an effective prescription for the prevention and treatment of recurrence and metastasis of postoperative GC at the Guang’anmen Hospital of China Academy of Chinese Medical Sciences, and explore its mechanisms.

## 2 Materials and methods

### 2.1 Design

The multicenter, randomized, double-blind, placebo-parallel-controlled clinical study will be conducted at seven hospitals, including Guang’anmen Hospital of the China Academy of Chinese Medical Sciences, Jiangsu Provincial Hospital of Traditional Chinese Medicine (TCM), the First Affiliated Hospital of Guangzhou University of Chinese Medicine, and Yueyang Hospital of Integrated Traditional Chinese and Western Medicine Affiliated to Shanghai University of Chinese Medicine. After signing the informed consent forms, 212 participants will be randomly assigned to the intervention group (YWJP) or the control group (YWJP placebo).

The key herbs of YWJP include *Astragalus membranaceus* (30 *g*)*, Codonopsis pilosula* (15 *g*)*, Angelica dahurica* (10 *g*)*, Curcuma zedoary* (9 *g*)*, Rhizoma nardostachyos* (10 *g*)*, Polygonum cuspidatum* (10 *g*)*, Radix Actinidiae chinensis* (15 *g*), and *Paris polyphylla* (9 *g*) (108 g in total). The control group will receive a YWJP placebo intervention comprising maltodextrin, lactose, bitters, citric acid, and other edible-grade raw materials (108 g in total). All experimental herbs and placebos used in this study will be provided by Jiangyin Tianjiang Pharmaceutical Co., Ltd. All raw materials have undergone safety assessments and quality inspection reports have been issued. YWJP and its placebo have been confirmed to be safe, reliable, controllable in quality, and similar in shape, color, smell, and taste.

**Figure 1:**
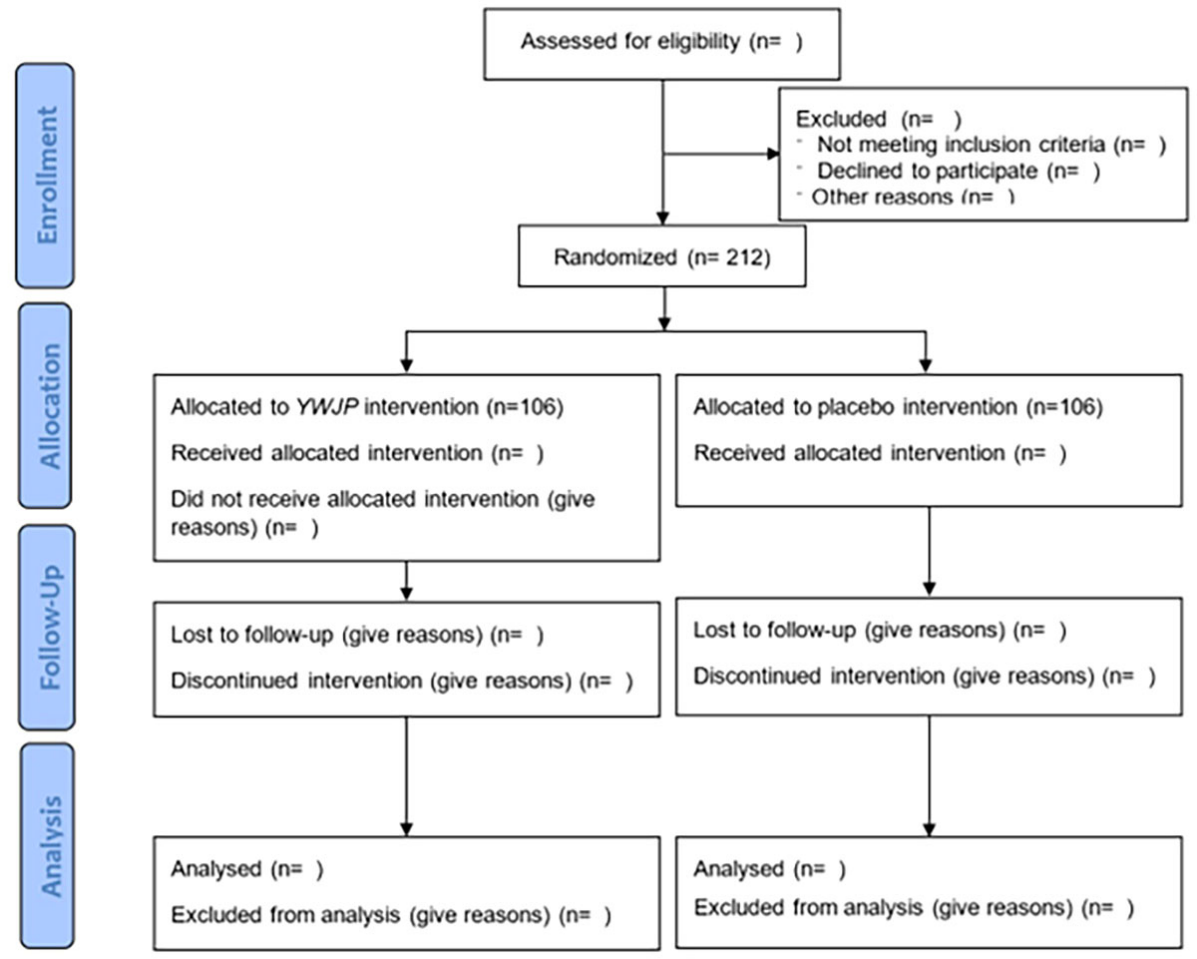
CONSORT flow diagram.

The primary and secondary outcomes will be evaluated immediately at the end of the follow-up period. The study process is illustrated in **Figure 1**. The clinical trial protocol complied with the Clinical Trial Standard Protocol Items: Recommendations for Interventional Trials (56) and Consolidated Standards of Reporting Trials (CONSORT) (57).

### 2.2 Recruitment

We plan to recruit patients from seven traditional Chinese oncology hospitals nationwide, led by Guang’anmen Hospital, and enroll patients by following up with expert clinics, playing recruitment advertisements on hospital LED screens, and posting recruitment posters. When we encounter potential patients, we will introduce our study protocol, including the objectives, intervention methods, processes, and potential adverse reactions. The research team will conduct repeated screenings of patients on a two-person basis. Patients who meet the inclusion criteria and wish to voluntarily participate in the study will sign an informed consent form.

### 2.3 Study population

The diagnostic criteria for GC, according to the 2021 Chinese Society of Clinical Oncology (CSCO), will be followed. The diagnosis and treatment of GC follow the clinical guidelines outlined in the 8th edition of the International Union Against Cancer (UICC) classification system (58).

### 2.4 Inclusion criteria

1. Research cases must be sourced from real-world registration platforms.
2. Stage II–III non-esophageal gastric junction GC that meets the diagnostic criteria and does not indicate tumor recurrence or metastasis by imaging.
3. Patients with GC who underwent radical gastrectomy (R0) within 8 months after surgery and completed at least six cycles of adjuvant chemotherapy with standard regimens (XELOX and SOX).
4. Eastern Cooperative Oncology Group (ECOG) performance status score of 0–2.
5. Patient ages ranging from 18 to 75 years, with no sex limitations.
6. Expected survival time ≥3 months.
7. Patients who voluntarily participated in the study, signed an informed consent form, and participated in the follow-up.

### 2.5 Exclusion criteria

1. Patients with concomitant primary tumors in other areas.
2. Patients with GC who were pathologically diagnosed with adenosquamous carcinoma, lymphoid interstitial carcinoma (medullary carcinoma), hepatoid adenocarcinoma, squamous cell carcinoma, signet ring cell carcinoma, undifferentiated carcinoma, and other gastric malignancies, such as gastric neuroendocrine tumors, gastric interlobular tumors, and gastric malignant lymphoma.
3. Patients who received neoadjuvant chemotherapy before surgery.
4. Patients who have been and are currently receiving targeted drug therapy.
5. Patients who have undergone or are currently undergoing gastric radiation therapy.
6. Patients who have undergone or are currently undergoing tumor immunotherapy.
7. Patients with mental illness.
8. Patients with severe and uncontrollable organic lesions or infections such as decompensated heart, lung, or kidney failure, who cannot tolerate chemotherapy.
9. Patients who underwent clinical trials of small-molecule drugs within 28 days or large-molecule drugs within 3 months.
10. Patients who are known to be allergic or intolerant to the study drug.

### 2.6 Criteria for withdrawal and removal

1. Those who experienced unexpected events during the treatment process and were unable to adhere to the protocol.
2. Patients who voluntarily requested withdrawal.
3. The researchers judged patients who exhibited poor compliance and were unable to continue clinical research.
4. Patients experiencing pregnancy, death, or loss of follow-up.

### 2.7 Randomization and blinding

This study will apply an Interactive Web Response System for central randomization and implement hidden allocation schemes. Patients will be randomly divided into intervention and control groups in a 1:1 ratio based on central randomization. The R software (V3.3.3) will be used to generate random sequences with three rounds of cyclic random statements. The blinding level will be double-blind, in which neither the researcher nor the participant have any idea of the specific details of the study. Anonymizing will be performed by statisticians who did not participate in the clinical trials and divided into two levels. This trial will establish a dedicated “emergency letter” for clinical trials that can only be urgently unblinded when the patient experiences an emergency. Handling this situation requires a clear understanding of the patient’s medication information.

### 2.8 Intervention

The intervention group will receive YWJP, whereas the control group will receive YWJP placebo. Patients will take one pack each time, dilute it with boiling water, and administer it twice daily (in the morning and evening). The course of treatment will be 4 weeks, and six courses are planned.

### 2.9 Criteria for adherence

After enrollment, patients will take their medication according to the method described in the “intervention” section. During the intervention, symptoms such as nausea, vomiting, liver and kidney dysfunction, diarrhea, and infection will be treated with medication. However, specific symptoms and combined medications must be recorded. Receiving modern antitumor treatment (including chemotherapy, immunotherapy, molecular targeted therapy, and radiotherapy) or taking other Chinese herbal decoctions, Chinese herbal injections, and traditional Chinese patent medicines with antitumor effects during the intervention will be prohibited.

### 2.10 Criteria for discontinuation

If unexpected adverse events occur, participants should no longer follow the study guidelines for such events. The investigator should comprehensively analyze whether these events are related to the experimental and control drugs used, and should decide whether to discontinue the clinical trial based on the participant’s condition. Patients who discontinue trials owing to serious adverse events should be followed up, and their outcomes should be documented. In addition, participants who could not adhere to the treatment and those who requested active withdrawal, had poor compliance, were pregnant, dead, or lost to follow-up met the criteria for discontinuation.

### 2.11 Sample size

This was a randomized controlled trial. The primary outcome was the 2-year disease-free survival (DFS) rate of patients with GC after surgery. It was estimated that the DFS rate 2 years after surgery in the control group was 43.9%, whereas the DFS rate in the intervention group was 63.9%. For α = 0.05 (bilateral), β = 0.2. Assuming that the enrollment rate of the study patients remained unchanged, the proportion of the intervention group to the control group was 1:1. The sample size for N1 was 96 cases in the intervention group, and for N2 was 96 cases in the control group, as calculated using the PASS 11 software. However, a detachment rate of 10% must be considered in the research process. Therefore, the intervention and control groups comprised 106 patients each (212 patients total).

### 2.12 Outcome measurements

The 2-year DFS rate as the primary outcome and DFS, OS, 1- to 3-year cumulative recurrence and metastasis rate, and 1- to 3-year cumulative survival rate as the secondary outcome will be calculated at the end of follow-up. The lymphocyte count-to-monocyte count ratio (LMR) (59), lymphocyte count-to-neutrophil count ratio (LNR) (60), prognostic nutritional index (PNI) (61), Quality of Life Questionnaire of Stomach 22 (QLQ-STO22) (62), M. D. Anderson Symptom Assessment Scale Gastrointestinal Tumor Specific Module (MDASI-GI) (63), Postgastrectomy Syndrome Assessment Scale-45 (PGSAS-45) (64) (diagnosis of the syndrome should refer to the 2014 *Guidelines of Diagnosis and Therapy in Oncology with Traditional Chinese Medicine*), and other secondary outcome indicators will be collected at weeks 0–6, whereas the total fat area (TFA) (65), visceral fat area (VFA) (66), subcutaneous fat area (SFA) (67), visceral adiposity index (VAI) (68), and tumor markers will be collected at weeks 0, 3, and 6. All measurement results will be recorded in the case report form (CRF). The details of outcome measurement projects are shown in **Table 1**.

**Table 1:** Treatment stage flowchart.

Item		Baseline	Treatment observation				Follow-up
Number of visits		1st time	2nd time	3rd time	4th time	*N*th time	*N* + *X*th time
Observation time		Days −7 to 0	Days 28–35	Days 56–63	Days 84–91	End of *N*th treatment course	Once every 3 months
Inclusion/Exclusion criteria		√					
Sign informed consent form		√					
Collect medical history							
Demographic data		√					
Diagnosis and TNM classification		√					
Past history and comorbidities		√					
Clinical observation							
Vital signs		√	√	√	√	√	√
Physical examination		√	√	√	√	√	√
Survival situation		√	√	√	√	√	√
MDASI-GI		√	√	√	√	√	√
Tongue and pulse condition		√	√	√	√	√	√
TCM syndrome		√	√	√	√	√	√
Imaging examination	PET-CT/CT/X-ray	√			√	√	√
	MRI, B-mode ultrasonography	*	*	*	*	*	*
	Bone scanning	*	*	*	*	*	*
	Gastroscope	*	*	*	*	*	*
Tumor marker		√	*	*	√	*	*
Indicators related to fat distribution		√	*	*	√	*	*
QLQ-STO22		√	√	√	√	√	√
PGSAS-45		√	√	√	√	√	√
Safety observation							
Blood routine		√	√	√	√	√	√
Hepatic and renal function		√	√	√	√	√	√
Urine and stool routine		*	*	*	*	*	*
Electrocardiogram		√	√	√	√	√	√
NCI adverse reaction			√	√	√	√	√
Adverse event			√	√	√	√	√
Other work							
Efficacy evaluation			√	√	√	√	√
Drug combination			√	√	√	√	√

*This check is optional.

√ This check is necessary.

#### 2.12.1 Primary outcome

The primary outcome of this study is the 2-year DFS rate after surgery, which refers to the proportion of patients who have not experienced recurrence, metastasis, or (for any reason) death within 2 years after surgery.

#### 2.12.2 Secondary outcome

1. Prognosis related indicators

DFS: Time from randomization to the onset of tumor progression or (for any reason) death in patients.

OS: Time from randomization to death from any cause.

Annual cumulative recurrence and metastasis rate for 1–3 years: The proportion of patients who experience recurrence and metastasis within 1–3 years from the day of surgery to the total number of patients.

Annual cumulative survival rate for 1–3 years: The proportion of patients with a survival period of 1–3 years or more from the day of surgery to the total number of patients.

2. Fat distribution-related indicators

TFA of the abdomen: A CT plain scan will be used to measure the fat area on cross-sectional images, directly reflecting the accumulation of abdominal fat in the human body. It is generally believed that the umbilical plane or L2/L3 gap can better reflect the body’s abdominal fat.

VFA: A commonly used indicator in clinical practice to evaluate the level of visceral fat. The precise measurement method is usually based on imaging methods, specifically the area occupied by adipose tissue in a certain section of abdominal CT (flat umbilical section or third lumbar section).

SFA: SFA = TFA − VFA.

VAI: This is another indicator for evaluating visceral adipose tissue accumulation and dysfunction, and is a new visceral fat assessment index calculated based on waist circumference (WC), body mass index (BMI), triglycerides (TG), and high-density lipoprotein (HDL).

Male VAI = [WC/(39.68 + 1.88 × BMI)] × (TG/1.03) × (1.31/HDL);

Female VAI = [WC/(36.58 + 1.89 × BMI)] × (TG/0.81) × (1.52/HDL).

3. Tumor markers: Carcinoembryonic antigen (CEA), carbohydrate antigen 724 (CA724), and carbohydrate antigen 199 (CA199) should be included as tumor markers.

4. Peripheral blood inflammatory indicators:

LMR: Ratio of lymphocyte count to monocyte count

LNR: Ratio of lymphocyte count to neutrophil count

5. Prognostic nutritional index (PNI): Record serum albumin (ALB) and lymphocyte (TLC) counts, with the formula PNI = ALB + 5 × TLC.

6. Symptoms and quality of life evaluation:

The QLQ-STO22 developed by the European Organization for Research and Treatment of Cancer will be used to evaluate the impact of treatment protocols on the quality of life of GC patients.

The impact of treatment regimens on patient symptoms will be evaluated using the MDASI-GI.

The quality of life with GC patients after gastrectomy will be measured using the PGSAS-45, and the intensity of various symptoms of post-gastrectomy syndrome will be understood.

7. Medication compliance: The number and percentage of cases will be calculated based on <80%, 80%–120%, and >120% medication compliance.

Medication compliance = actual dosage/expected dosage × 100% (rounded to two decimal places).

8. Adverse reaction rate: The proportion of adverse reactions caused by drugs in the enrolled population.

### 2.13 Safety evaluation

Safety evaluations were performed according to the National Cancer Institute’s Common Terminology Standard for Adverse Events (CTCAE v.5.0), and patient adverse events were monitored every 3 months from baseline to disease progression, death, or 2 years after surgery. The evaluation methods included routine blood tests, urine tests, biochemical tests, and electrocardiography.

### 2.14 Data collection and management

Patients will undergo periodic follow-up (once a month during the treatment period and once every 3 months during the follow-up period, including electronic questionnaires or telephone follow-up). After surgery, they will be observed for at least 3 years. Data on vital signs, physical examination, weight, height, body surface area, BMI, ECOG score, KPS score, QLQ-STO22, MDASI-GI, PGSAS-45, peripheral blood inflammatory indicators, blood and urine routine, complete biochemical tests, electrocardiogram, and blood tumor markers will be collected for each treatment cycle and during follow-up visits every 3 months. For participants who did not experience disease progression after completing six courses of treatment, imaging follow-up was conducted every 3 months for 2 years post-surgery and then every 6 months after that until disease recurrence or initiation of alternative therapies. The basic information of the patients and the relevant information required for the study will be recorded in the CRF. Only authorized researchers, representatives of research-undertaking units, ethics committees, and higher-level management departments can access patient records upon reasonable request. No public reports of the results of this study disclose the patient’s name or identity. The research team will protect the privacy of the patient’s medical data as much as possible within the scope permitted by law.

### 2.15 Quality control

This study will introduce and promote an ISO quality management system. Personnel at all levels will receive the necessary training in management and quality awareness. A quality control system will be established during project implementation, and relevant quality control measures and evaluation plans will be formulated. The project lead unit will assign special personnel to conduct quality control and supervision of this study, including clinical data collection standards and data verification quality control measures. In addition, the project involves task verification and quality control of participating units to ensure completion. A quality-verification document was created and stored for archival purposes. In addition, Guang’anmen Hospital of the China Academy of Chinese Medical Sciences selected a third party to establish the clinical database of the participants and conducted a statistical analysis of the data.

### 2.16 Statistical analysis and method

SAS 9.4 statistical software will be used, and full analysis set (FAS) and protocol compliance set (PPS) analyses will be performed on the efficacy indicators. A safety dataset analysis should be conducted for adverse reactions. All statistical tests will be conducted bilaterally, and statistical significance will be set at *p* ≤ 0.05. We will analyze whether there are outliers in the data and conduct a professional analysis of outliers to decide whether to accept or reject them. After this, we will analyze the data for missing values and conduct a professional analysis to determine whether the missing values are listed as missing or data transferred. The proportion of shedding cases should not exceed 10%; otherwise, analysis and explanation should be provided. The measurement data were described as mean, standard deviation, median, minimum, and maximum whereas counting data were described as frequency, percentage, etc. Quantitative data analysis will be performed using *t*-tests, rank-sum tests, etc. Counting data analysis will be performed using chi-square tests, ridit analyses, etc. Survival data analysis will be performed using the Kaplan–Meier method, Wilcoxon rank sum test, or log-rank test. A Cox-proportional risk regression model was used for multivariate survival analysis.

## 3 Discussion

Gastric cancer is an important component of the global cancer burden, and local recurrence or distant metastasis after radical gastrectomy is the primary cause of poor prognosis (26). The guidelines recommend no standard treatment after postoperative adjuvant treatment for GC; watchful waiting is recommended. However, most patients with GC experience recurrence and metastasis within 2–3 years after surgery. Therefore, exploring the efficacy of complementary and alternative medicines during surveillance and watchful waiting periods is necessary to promote adjustment of postoperative monitoring and management programs. Several studies have shown that TCM plays a significant role in preventing and treating GC. TCMs stabilize tumors, reduce recurrence and metastasis rates, alleviate clinical symptoms, improve patient survival and quality of life, and decrease the occurrence of adverse reactions (69, 70). However, the quality of most clinical research on TCM treatment of GC is low. These studies provide a low level of evidence, making it challenging to effectively promote and guide clinical practice. Moreover, no large-sample study has explored the correlation between *Yang* deficiency syndrome and the postoperative recurrence and metastasis of GC. Carrying out well-designed, high-quality clinical research and obtaining robust evidence-based data on TCM treatment for GC are key challenges hindering the widespread adoption of TCM in treating GC. A meta-analysis (71) demonstrated that integrated Chinese and Western medicine treatment could decrease the recurrence rate of GC at 12, 24, and 36 months postoperatively. It was significantly superior to Western medicine treatment alone. *Fuzheng Jiedu* is an effective treatment for postoperative recurrence and metastasis of GC that is used in the Oncology Department of Guang’anmen Hospital of the China Academy of Chinese Medical Sciences. Studies have shown (52) that the addition of the *Fuzheng Jiedu* prescription (predecessor of the YWJP) for patients with GC can reduce the recurrence and metastasis rates of GC for 2 years after surgery by 18.60%, which is approximately a 25% reduction compared to patients in the same period. Therefore, TCM has therapeutic advantages in reducing the recurrence and metastasis of GC.

The YWJP has the effects of nourishing *Qi* and warming *Yang*, strengthening *Zhengqi*, and detoxifying, which can improve the “*Yang-deficient toxic node*” status of postoperative patients with GC. In this study, we aim to clarify the efficacy and safety of YWJP in preventing and treating postoperative recurrence and metastasis of GC. Furthermore, we aim to explore the correlation between *Yang* deficiency, metabolic abnormalities, gut microbiota, and other factors and their impact on long-term prognosis. Lastly, we seek to identify the mechanisms of postoperative recurrence and metastasis of GC under the guidance of core pathogenesis and possible targets of TCM. The advantages of this study are as follows: (1) it is a high-quality, randomized, double-blind, controlled, multicenter clinical study with a 1:1 random allocation. This design will ensure a better balance between groups and effectively avoid the impact of potentially unknown factors on the test results. Furthermore, simultaneously observing both groups of patients helps to avoid the effect of the trial sequence on the results, thereby enhancing the credibility of the research findings. (2) This research provides a solid clinical foundation for the initial phase. It is centered on the core pathogenesis of the “*Yang-deficient toxic node*” following surgery for GC. The treatment approach of “supplementing *Qi* and warming *Yang* and detoxify” aims to guide personalized treatment strategies aligned with the principles of precision medicine, focused on the effective intervention effect on GC recurrence and metastasis. However, this study has limitations: (1) The experimental design and implementation requirements are demanding. The intervention time is lengthy, the workload is substantial, and the implementation is challenging. (2) The intervention and follow-up periods were lengthy, with a high probability of dropout. (3) Considering the limitations of manpower, economy, and time involved in this clinical study, our follow-up time will be set at 3 years after surgery. However, we will continue to monitor this study to compensate for the restricted follow-up time.

The aims of this study were to evaluate the effectiveness and safety of supplementary and alternative drugs (YWJP) for recurrence and metastasis in patients with GC after surgery and to reveal the influence of *Yang* deficiency syndrome and gut microbiota on recurrence and metastasis. In addition, based on the core pathogenesis of the *Yang-deficient toxic node*, exploring the preventive and therapeutic effects and molecular mechanisms of TCM after GC surgery will establish a foundation for developing a new theoretical framework for preventing and treating GC post-surgery. Ultimately, the results of this study will provide a crucial foundation for patients with GC, clinicians, and policymakers to monitor postoperative treatment management and establish a standardized treatment protocol. In the future, we will promote the clinical transformation of YWJP, develop new Chinese medicines, and conduct high-quality, multicenter, randomized controlled studies internationally to enhance the standardized clinical diagnosis and treatment of GC. In addition, with the advancement of spatial tumor ecology (72, 73), it is crucial to investigate the biological implications of the “disease-symptom-syndrome” of GC and the biological foundation of the population that benefits from TCM prevention and treatment.

## Data availability statement

The original contributions presented in the study are included in the article/supplementary material. Further inquiries can be directed to the corresponding author.

## Ethics statement

This study has been performed in accordance with the Declaration of Helsinki and has been approved by ethics Committee of Guang 'anmen Hospital, China Academy of Chinese Medical Sciences (Ethics Approval Number: 2021-147-KY). Written informed consent was obtained from the individual(s) for the publication of any potentiallyidentifiable images or data included in this article.

## Author contributions

LC: Writing – original draft, Writing – review & editing, Data curation, Methodology. GZ: Methodology, Writing – review & editing. XW: Methodology, Supervision, Writing – review & editing. ZK: Methodology, Writing – review & editing. XS: Supervision, Writing – review & editing. XM: Supervision, Writing – review & editing. XZ: Methodology, Writing – review & editing. RG: Supervision, Writing – review & editing. JL: Supervision, Writing – review & editing.
